# Physicochemical properties of flowable composites using isobornyl methacrylate as diluent monomer

**DOI:** 10.1590/1678-7757-2024-0172

**Published:** 2024-09-20

**Authors:** Roberta Pinto PEREIRA, Dayane de OLIVEIRA, Mateus Garcia ROCHA, Lourenço CORRER-SOBRINHO, Jean-François ROULET, Mario Alexandre Coelho SINHORETI

**Affiliations:** 1 Universidade Estadual de Campinas Faculdade de Odontologia de Piracicaba Departamento de Odontologia Restauradora Piracicaba SP Brasil Universidade Estadual de Campinas, Faculdade de Odontologia de Piracicaba, Departamento de Odontologia Restauradora, Piracicaba, SP, Brasil.; 2 University of Florida College of Dentistry Department of Restorative Dental Sciences Gainesville FL United States University of Florida, College of Dentistry, Department of Restorative Dental Sciences, Gainesville, FL, United States.

**Keywords:** Dental materials, Methacrylate, Dental resin, Monomers

## Abstract

**Objective:**

this study sought to evaluate the effect of isobornyl methacrylate (IBOMA) as a diluent monomer on the physicochemical properties of experimental flowable resin composites.

**Methodology:**

the organic resin matrix of a modal flowable resin composite was formulated with 50 wt.% of bisphenol-A-glycidyl methacrylate (Bis-GMA) and 50 wt.% of a diluent monomer, in which IBOMA was used as a combining or substituent diluent monomer to triethylene glycol dimethacrylate (TEGDMA). The resin matrices were filled with 55 wt.% particles, of which 10 wt.% was 0.05-μm fumed silica, and 45 wt.% was 0.7-μm BaBSiO2 glass. Polymerization shrinkage stress (PSS; n=10), degree of conversion (DC; n=3), maximum rate of polymerization (Rpmax; n=3), film thickness (FT; n=10), sorption (Wsp; n=10), solubility (Wsl; n=10), flexural strength (FS; n=10), flexural modulus (FM; n=10), Knoop microhardness (KH; n=10), and microhardness reduction after chemical softening (HR; n=10) were evaluated. Data were analyzed using one-way ANOVA, followed by Tukey’s test (α=0.05; β=0.2).

**Results:**

the results showed that the substitution or addition of IBOMA reduced FT (p=0.001), PSS (p=0.013), Rpmax (p=0.001), DC (p=0.001), FM (p=0.006) Wsp (p=0.032), and Wsl (p=0.021). However, when used as a complete substituent, IBOMA demonstrated significantly lower FS (p=0.017) and KH (p=0.008), while TEGDMA demonstrated significantly lower HR (p=0.022).

**Conclusion:**

the flowable composite containing IBOMA combined with TEGDMA showed no effect in KH and FS and effectively reduced the PSS, RP, FT, Wsp, and Wsl. However, it showed a reduction in DC, FS, and an increase in HR.

## Introduction

Most commercially available resin composites are prepared with methacrylate monomers, which provide high mechanical strength, low volatility, and relatively low shrinkage stress.^1,2^ Shrinkage stress at the adhesive layer can result in failure of direct and indirect restorations, as well as gap formation, microleakage, and marginal staining.^3-7^ Although a high degree of conversion (DC) is essential for withstanding masticatory forces and chemical degradation of resin-based materials,^8^ it can lead to a high modulus of elasticity and volumetric shrinkage, both of which are associated with shrinkage stress.^9^

Bisphenol-A-glycidyldimethacrylate (BisGMA) is one of the most commonly used monomers in flowable resin composites due to its satisfactory mechanical properties, degree of conversion, and rheological properties.^10^Because of its high viscosity, diluent monomers need to be added to the resin material matrix to improve handling at room temperature,^11-12^ to allow the incorporation of inorganic fillers, to increase the monomer’s mobility during the polymerization reaction, and to achieve higher DC.^13^ Low viscosity dimethacrylate monomers, such as triethylene glycol dimethacrylate (TEGDMA) are often added to flowable composites to increase flowability and decrease film thickness.^13-14^Despite its optimal diluent ability, TEGDMA decreases mechanical strength and increases volumetric shrinkage^7^ and water sorption of resin-based materials.^15^ Thus, alternative diluent monomers have been investigated as replacements for TEGDMA.

Isobornyl methacrylate (IBOMA), a monomethacrylate monomer, is known to reduce volumetric shrinkage, water solubility, and solvent degradation of resin blends.^7,16^ Due to its low viscosity, low volumetric shrinkage, and high hydrophobicity,^17^IBOMA has been used for synthesizing nanogel, which is added to the resin composite matrix to reduce volumetric shrinkage, shrinkage stress, water absorption, and chemical degradation.^18,19^ Used as a diluent monomer in flowable resin composites, IBOMA could reduce the volumetric shrinkage, film thickness, sorption, and solubility and thus increase the longevity of the restorations.

In this context, isobornyl methacrylate (IBOMA) emerges as a promising alternative, as it offers reduced volumetric shrinkage, water solubility, and solvent degradation alongside enhanced hydrophobicity and decreased viscosity. These properties suggest that IBOMA could significantly extend the longevity and improve the effectiveness of dental restorations by mitigating the adverse effects associated with traditional diluent monomers. Therefore, the present study aimed to evaluate the effect of IBOMA as an alternative diluent monomer on the physicochemical properties of modal flowable resin composites. The alternative hypotheses were that: (i) IBOMA, used by itself or (ii) combined with TEGDMA as a diluent monomer, would have no significant negative effect on the physicochemical properties of experimental resin composites.

## Methodology

### Flowable resin composite formulation

**Figure 1:**
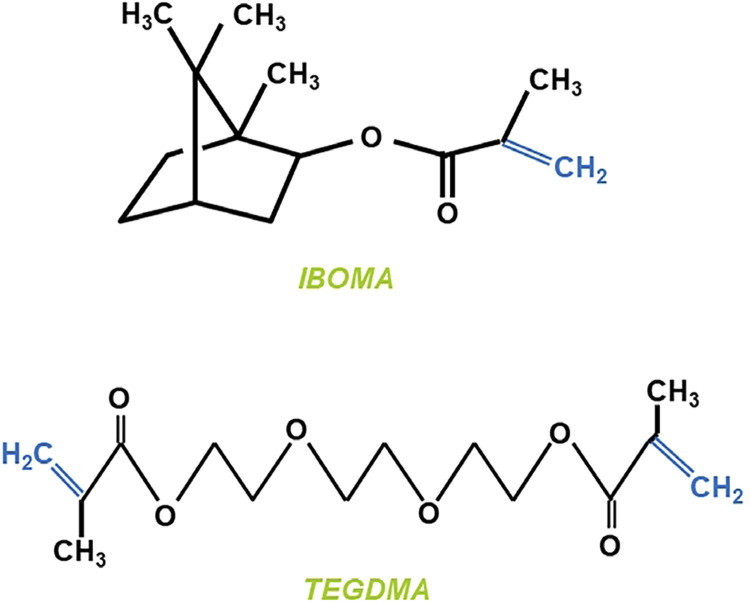
Molecular framework of isobornyl methacrylate (IBOMA) and triethylene glycol dimethacrylate (TEGDMA).

Three experimental flowable resin composites were mechanically mixed using a centrifugal mixing device (SpeedMixer, DAC 150.1 FVZ- K, Hauschild Engineering, Hamm, North Rhine-Westphalia, Germany). The organic resin matrix of the experimental composites included 50 wt.% Bis-GMA (Sigma-Aldrich Inc., St Louis, MO, USA) and 50 wt.% of a diluent monomer, in which IBOMA (Sigma-Aldrich Inc., United States) was used as a combining or substituent diluent monomer to triethylene glycol dimethacrylate (TEGDMA) (Sigma-Aldrich Inc., USA). Figure 1 illustrates the chemical structure of both diluent monomers. The photoinitiator system in all formulations was composed of 0.5 wt.% camphorquinone (Sigma-Aldrich Inc., USA) combined with 1 wt.% 2-(dimethylamino)ethyl methacrylate (Sigma-Aldrich Inc., USA). Then, it was loaded with 55 wt.% of filler particles, in which 10 wt.% was 0.05 μm fumed silica (Aerosil OX50, Nippon Aerosil Co. Ltd., Yokkaichi, Tokyo, Japan) and 45 wt.% was 0.7-μm BaBSiO_2_ glass (Esstech Inc., Essington, PA, United States).

Formulation 1 (50% BisGMA, 50% TEGDMA):

The molar ratio of BisGMA to TEGDMA is approximately 1:1.79.

Formulation 2 (50% BisGMA, 50% IBOMA):

The molar ratio of BisGMA to IBOMA is approximately 1:2.46.

Formulation 3 (50% BisGMA, 25% TEGDMA, 25% IBOMA):

The molar ratio of BisGMA to TEGDMA to IBOMA is approximately 1:0.90:1.23.

### Polymerization shrinkage stress (PSS) test

The polymerization shrinkage stress (PSS) was measured (n=10) using a universal testing machine (Instron 4411, Instron, Canton, MA, USA).^8,20^ The PSS value for each specimen was calculated considering the maximum nominal strength (N) and the displacement (µm) created by the flowable composite after photoactivation. Two glass rods (4 mm in diameter and 13 and 54 mm in length) had their ends roughened with #180-grit sandpaper (Buehler, Lake Bluff, IL, United States) and were connected to the universal testing machine (Instron 4411, Instron, Canton, MA, USA). The upper portion of the rod was connected to the machine’s load cell in a 26-mm slot, while the lower portion was connected to a base containing a hole for the curing light to reach the rod surface. The end of each rod in contact with the composite was previously etched for 10 s using 10% hydrofluoric acid (Dentsply Sirona, São Paulo, Brazil) and cleaned with moist cotton. A single layer of silane agent (Monobond Plus, Ivoclar Vivadent, AG, Schaan, Liechtenstein) was applied to both surfaces. The rods were vertically aligned 1 mm apart.

The flowable composite was then applied to the 13-mm rod, keeping a 1-mm space between the rods. The radiant emittance was measured (1200 mW/cm^2;^Valo cordless, Ultradent, St Louis, MO, USA), and a reduction of 20% (960 mW/cm^2^) was detected after the light passed through the lower rod; therefore, the photoactivation time was set at 25 s to contemplate a total of 24 J/cm^2^of radiant exposure.^21^ A video extensometer, consisting of a DSLR camera (Canon t3i, Melville, NY, USA), a 100-mm macro lens (Canon, United States), and displacement calculation software (Trackmate, Fiji, ImageJ, National Institute of Health, Bethesda, MD, USA), was used to measure the displacement (µm) of the rods.^8^ Based on the displacement data (µm) obtained from three images recorded before and after placement of the flowable composite and after photoactivation. The testing system compliance (1.66 µm/N) was calculated considering a C-factor of 2.^22^ The resin composite strain value provided by the video extensometer was assessed using a universal testing machine, considering compliance of 1.66 µm/N for the apparatus feedback system and another of 0.4 μm/N (C-factor 2) related to Class I cavity.^22^ A previously published formula^23^ was used to calculate the maximum PSS (MPa) by summing the nominal (PS_nominal_) and corrected (PS_corrected_) polymerization stress values and dividing the sum by the cross-sectional area of the glass rod.^24^

### Polymerization kinetics

The polymerization kinetics (n = 3) was analyzed using an ATR-FTIR spectrometer (Nicolet iS20, Thermofisher, Waltham, MA, USA) to measure the degree of conversion (DC) and maximum rate of polymerization (Rp_max_). The unpolymerized flowable resin composites were placed on the diamond ATR detector of the FTIR spectrometer, scanned, and then light-cured (Valo cordless, Ultradent, USA) at a radiant emittance of 1200 mW/cm^2^ for 20 s. The polymerized flowable resin composites were scanned again 300 s after light curing. The unconverted carbon double bonds were quantified by calculating the ratio derived from the aliphatic C=C (vinyl) absorption area (1638 cm^1^) to the aromatic C=C absorption area (1608 cm^1^) signals for both polymerized and unpolymerized specimens. Absorbance spectra included 32 scans at a resolution of 4 cm^1^. The DC for the flowable resin composites was calculated according to the following equation:


$$DC(\%)=\left(1-\frac{Xa/Ya}{Xb/Yb}\right)\times 100$$

Where Xa (polymerized) and Xb (unpolymerized) represent the bands of the polymerizable aliphatic double bonds, and Ya (polymerized) and Yb (unpolymerized) represent the bands of the aromatic double bonds.

The maximum rate of polymerization (Rp_max_) was obtained at the first derivate of the degree of conversion versus time.

### Film thickness (FT) test

The film thickness (n=10) test was done in compliance with ISO 4049:2019.^25^ Two glass plates with a contact surface area of 200 mm^2^ were placed together, and a digital micrometer (Mitutoyo, Tokyo, Japan) was used to measure their thickness four times. A standardized volume (0.10 mL) of each flowable resin composite was then inserted between the plates — with a 150-N load applied to the upper plate for 180 s — and then photoactivated for 20 s under 1200 mW/cm^2^ of radiant emittance (Valo cordless, Ultradent, USA). Subsequently, four additional thickness measurements were taken. The film thickness was calculated by deducting the average of the initial and final measurements with and without the different flowable resin composites interposed.

### Water sorption (Wsp) and solubility (Wsl) tests

The W_sp_ and W_sl_ tests were conducted in compliance with ISO 4049:2019.^25^ Ten cylindrical specimens (1 mm thick × 15 mm in diameter) were prepared. Initially, the center area (10 mm in diameter) of each specimen was light-cured (Valo cordless, Ultradent, USA) at 1200 mW/cm^2^ for 20 s. Then, another eight areas (10 mm in diameter) around the center area were selected and light-cured at 1200 mW/cm^2^ for 20 s each, while ensuring the nine areas overlapped each other. Light curing procedures were done on both sides of the specimens (top and bottom), which were then stored in desiccators containing silica gel at 37 °C.

The specimens were weighed every 24 hours using a 0.001-g accuracy analytical balance (Tel Marke, Bel Quimis, São Paulo, SP, Brazil). The weighing cycle was repeated until a constant mass (m_1_) was obtained (no weight change for 2 days). The thickness and diameter of each specimen were measured using a digital electronic caliper (Mitutoyo Corporation, Tokyo, Japan). These values were used to calculate the volume (V) of each specimen (mm^3^). The specimens were stored in plastic containers with distilled water (6 mL per specimen) at 37 °C for 7 days and then dried with absorbent paper and weighed again until the constant mass (m_2_) was obtained. The specimens were then desiccated again; the entire mass reconditioning cycle was repeated, and the constant mass (m_3_) was recorded. The values (μg/mm^3^) for W_sp_ and W_sl_ were calculated using the following equations:


$$W_{sp}=\left(m_{2}-m_{3}\right)/V$$

$$W_{s1}=\left(m_{1}-m_{3}\right)/V$$

### Flexural strength (FS) and flexural modulus (FM) tests

A three-point bending test (20-mm span) was used to measure the FS and FM of the flowable resin composites. Bar-shaped specimens (25×2×2mm; n=10) were fabricated according to ISO 4049:2019^25^ using a stainless-steel mold. The flowable composite was placed in the mold and light-cured (Valo cordless, Ultradent, USA) with the light tip in contact with the polyester strip on the specimens’ top surface at 1200 mW/cm^2^for 20 s. Due to the length (25 mm) of the specimens and the ranging area (10 mm in diameter) of the curing light, five overlapping irradiation cycles were carried out along the top and bottom sides of each specimen. After being stored in water at 37 °C for 24 h, the specimens were tested for FS and FM using a universal testing machine (Instron, model 4411, Canton, MA, USA) at a crosshead speed of 0.5 mm/min until failure. FS was expressed in megapascal (MPa) and FM in gigapascal (GPa) using the following equations:


$$FS=\frac{3\times L\times D}{2\times W\times h^{2}}\;\text{ and }\;FM=\frac{L\times D^{3}}{4\times W\times h^{3}\times d}\times 10^{-3}$$

Where L refers to the maximum load (N) at failure, D to the distance (span) between the rods, W to the specimen’s width, h to the specimen’s height, and d to the crosshead displacement.

### Knoop microhardness (KH) test

A stainless-steel mold was used to fabricate the flowable resin composite specimens (6 mm in diameter and 2 mm thick; n=10). The top surface of the specimens was covered with a polyester strip, and the excess of flowable composite was removed by pressing a glass slide against the mold. Each specimen was light-cured at 1200 mW/cm^2^ for 20 s using an LED curing unit (Valo cordless, Ultradent, USA). After light curing, the top surface of the specimens was polished with 1200- and 2000-grit silicon carbide paper under running water. After dry-storage at 37 °C for 24 h, the specimens had three indentations made on their top surfaces using a Knoop indenter (HMV-2, Shimadzu, Tokyo, Japan) under a load of 50 gf for 15 s. The KH mean value of each surface was obtained from the values of the three indentations.

### KH reduction (HR) test

The HR test (in ethanol) was used to indirectly verify the crosslink density of the experimental flowable resin composites. The KH specimens were stored in 100% ethanol at 37 °C for 24 h in the absence of light. The KH of each specimen was measured, and HR was determined by subtracting the initial (before ethanol storage) from the final (after ethanol storage) KH values.

### Statistical analyses

A power analysis, considering a power of at least 0.8, was previously carried out using data obtained from a pilot test to determine the sample size for each test. Data were checked for normality using Shapiro-Wilk’s test and for homoscedasticity using Levene’s test, at a significance level of α = 0.05 and *β* = 0.2. Data concerning PSS, DC, Rp_max_, FT, W_sp_, W_sl_, KH, HR, FS, and FM were analyzed using the one-way analysis of variance (ANOVA) in which the factor was set as the flowable resin composite diluent in three levels (IBOMA, TEGDMA, and TEG-IBO). Tukey’s test was applied for multiple comparisons of the groups considering all tests (α=0.05; *β*=0.2).

## Results

**Figure 2:**
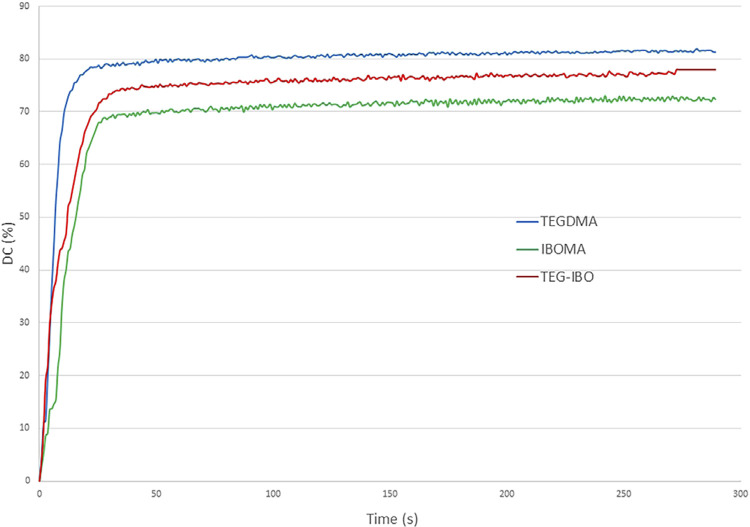
Degree of conversion (%) of the flowable resin composites over a period of 300 s.

**Figure 3:**
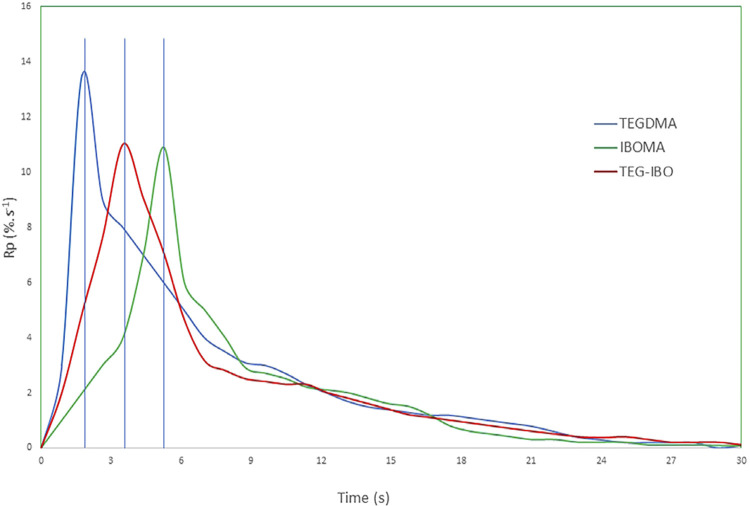
Maximum rate of polymerization (Rpmax: %. s-1) over a period 30 s.

IBOMA (p=0.013) and TEG-IBO (p=0.017) showed a significantly lower PSS when compared to TEGDMA (Table 1). DC was statically different among all groups (p=0.001), with TEGDMA showing the highest mean value (Table 1). The DC of the flowable resin composites (Figure 2) stabilized at 20 s (TEGDMA) and 25 s (IBOMA and TEG-IBO). IBOMA and TEG-IBO showed Rp_max_ significantly lower (Table 1) than that obtained for TEGDMA (p=0.016). The peak of Rp_max_ concerning TEGDMA, TEG-IBO, and IBOMA occurred at 2–3, 3–4, and 5–6 s, respectively (Figure 3). The FT obtained for IBOMA was significantly lower than that observed for TEG-IBO, whose mean value was significantly lower than that of TEGDMA (p=0.001).

**Table 1:** Mean values and standard deviation regarding polymerization shrinkage stress (PSS), degree of conversion (DC), maximum polymerization rate (Rpmax), and film thickness (FT) of the resin composites.

GROUPS	PSS (MPa)	DC (%)	Rp_**max**_ (%.s^**-1**^)	FT (µm)
TEGDMA	5.62 ± 1.7^A^	81.9 ± 0.3^A^	13.5 ± 0.9^A^	53.0 ± 0.4^A^
IBOMA	3.75 ± 1.1^B^	73.4 ± 0.5^C^	11.0 ± 0.5^B^	50.2 ± 0.4^C^
TEG-IBO	3.82 ± 1.1^B^	78.0 ± 0.2^B^	11.1 ± 0.7^B^	51.60 ± 0.4^B^

Different upper-case letters indicate statistical difference in columns (p<0.05).

Mean values concerning W_sp_, W_sl_, FS, and FM are shown in Table 2. IBOMA and TEG-IBO showed W_sp_ (p=0.032) and W_sl_ (p=0.021) significantly lower than those obtained for TEGDMA. FS for IBOMA was significantly lower than those for TEGDMA and TEG-IBO (p=0.017). FM for TEGDMA was significantly higher than those obtained for TEG-IBO and IBOMA (p=0.006), while no significant difference was observed between TEG-IBO and IBOMA (p=0.152).

**Table 2:** Means and standard deviation of the water sorption (Wsp), water solubility (Wsl), flexural strength (FS), and flexural modulus (FM) results for the tested resin composites.

GROUPS	W_**sp**_ (μg/mm^**3**^x10^**4**^)	W_**sl**_ (μg/mm^**3**^x10^**4**^)	FS (MPa)	FM (GPa)
TEGDMA	1.209 ± 0.100^A^	0.494 ± 0.064^A^	113.0 ± 16.5^A^	5.23 ± 0.83^A^
IBOMA	0.968 ± 0.166^B^	0.226 ± 0.059^B^	72.1 ± 9.4^B^	3.40 ± 0.71^B^
TEG-IBO	0.951 ± 0.087^B^	0.238 ± 0.052^B^	100.8 ± 7.8^A^	4.12 ± 0.36^B^

Different capital letters indicate statistical difference in columns (p<0.05).

The KH and HR of the flowable resin composites are shown in Table 3. The KH obtained for TEGDMA was significantly higher than that for IBOMA (p=0.008), with TEG-IBO showing no significant difference from the other flowable composites. The HR obtained for TEGDMA was significantly lower than those for IBOMA and TEG-IBO (p=0.008).

**Table 3:** Mean values and standard deviation of Knoop microhardness (KH) and hardness reduction (HR) of the experimental flowable resin composites.

GROUPS	KH	HR
TEGDMA	50.2 ± 10.5^A^	40.6 ± 19.8^B^
IBOMA	32.2 ± 1.7^B^	70.0 ± 8.7^A^
TEG-IBO	42.7 ± 8.3^AB^	66.6 ± 16.6^A^

Different capital letters indicate statistical difference in columns (p<0.05).

## Discussion

The first null hypothesis that IBOMA, used by itself as a diluent monomer, would have no significant negative effect on the physicochemical properties of the flowable resin composites was rejected. Despite reducing PSS, FT, W_sp_, W_sl_ and Rp_max,_ IBOMA significantly reduced DC, KH, FS and FM and increased HR. However, when combined with TEGDMA, IBOMA significantly reduced DC, PSS, FT, W_sp_, W_sl_, Rp_max_, FM, and HR and showed intermediate mean values of FS and KH. Therefore, the second hypothesis that IBOMA, combined with TEGDMA as a diluent monomer, would have no significant negative effect on the physicochemical properties of the flowable resin composites was partially accepted.

The kinetics of the polymerization reaction can influence the vitrification point, but not directly identify it.^7^ Delays in the vitrification process and reductions in PSS have been reported for IBOMA, which is a monomethacrylate monomer.^16^ IBOMA and TEG-IBO showed significantly lower Rp_max_ mean values (Table 1) as well as a delay in reaching Rp_max_, when compared to TEGDMA (Figure 3), which might have contributed to their lower PSS mean values. When compared with IBOMA, TEGDMA has more methacrylate groups for chemical bonds,^16^ which helps to explain the higher Rp_max_ values obtained for TEGDMA. Figure 1 illustrates the methacrylate groups from both methacrylate monomers, IBOMA and TEGDMA, highlighted in blue. It is noteworthy that TEGDMA has two methacrylate functional groups, while IBOMA only has one. The higher the Rp_max_, the greater the conversion of monomers prior to vitrification,^26^ which is a condition that might account for the higher DC mean value obtained for TEGDMA compared to that of IBOMA (Table 1). Moreover, as a monomethacrylate monomer, IBOMA does not contribute to polymer crosslinking, thus also contributing to a possible delay in the vitrification process and reducing shrinkage, as observed in the results.

The fact that IBOMA does not contribute to polymer crosslinking also explains the drastic drop in mechanical properties observed in the flowable composite in which IBOMA was used as the only diluent monomer. This also explains the lower KH, FS, FM, and consequently, higher W_sp_ and W_sl_ of IBOMA as the diluent monomer compared to TEGDMA. The addition of TEGDMA seems to be the primary factor contributing to the enhanced mechanical properties observed in the TEG-IBO group, resulting in comparable outcomes with the TEGDMA group. While the aliphatic ring structure of IBOMA might have a reinforcement effect, this impact would indeed be more pronounced in the IBOMA composites if it were the dominant factor. Therefore, the comparable mechanical properties between TEG-IBO and TEGDMA seem to be attributed primarily to the presence of TEGDMA, which facilitates higher crosslink density and mechanical strength.^27,28^

The aliphatic ring structure of IBOMA (as Illustrated in Figure 1) also influences the polymer’s physical properties by limiting the mobility and accessibility of reactive sites. This restriction can lead to a decrease in the rate of polymerization, as evidenced by the reduced polymerization shrinkage stress observed with IBOMA. Conversely, the TEGDMA’s linear structure, characterized by two methacrylate functional groups, allows for a higher density of cross-linking within the polymer matrix.^1^ These additional reactive sites favor faster polymerization rates, which contributes to a higher degree of conversion and increased shrinkage stress. The methacrylate groups of TEGDMA engage in more efficient chemical bonding, leading to a network with enhanced mechanical properties and crosslink density.^1^ Thus, although the bicyclic structure of IBOMA contributes to its reduced polymerization shrinkage and enhanced hydrophobicity, it also influences its ability to form linear polymers rather than cross-linked networks.^7^ This can lead to variations in mechanical properties, such as flexural strength and microhardness, by limiting the density of cross-links that contribute to the material’s rigidity and resistance to deformation.

This interplay between IBOMA’s restrictive ring structure and TEGDMA’s conductive linear configuration evidences the importance of molecular architecture in dictating the physicochemical properties of flowable resin composites.^7^ The complementary characteristics of IBOMA and TEGDMA suggest that their combination can be strategically planned to achieve a balance between reducing polymerization shrinkage stress while maintaining or enhancing some specific mechanical properties of different resin-based materials, depending on their indication.^16^

For direct restorative materials, mechanical properties such as strength, modulus of elasticity, and wear resistance, are important due to their direct exposure to the oral environment’s mechanical forces, such as chewing and grinding. These properties ensure the restoration can withstand function over time. Conversely, for resin-based materials used in cementing indirect restorations, while mechanical properties hold some importance, other properties such as film thickness, bond strength, and polymerization shrinkage stress are priorities. The thin film ensures a tight and accurate fit of the restoration, which minimizes the risk of microleakage, secondary caries, and restoration failure.^29^ This distinction highlights the nuanced approach needed in formulating resin-based materials, in which the intended application drives the prioritization of specific physicochemical properties.

Both IBOMA and TEG-IBO showed FT mean values significantly lower than that obtained for TEGDMA (Table 1). The lower viscosity (8.1 cp) and lower molecular weight (222.32 g/mol) of IBOMA, when compared with those of TEGDMA^16^ (9.15 cp and 286.32 g/mol, respectively) may have contributed to the low FT mean values obtained for IBOMA and TEG-IBO. However, although IBOMA shows lower viscosity than TEGDMA due to its lower molecular weight, its non-linear configuration caused by the presence of a bicyclic compound side chain (Figure 1) contributes to slow mobility/diffusion during the polymerization.^28^ Moreover, TEGDMA’s chemical structure may promote different intermolecular interactions with fillers than that of IBOMA, which potentially influences the dispersion of fillers within the matrix and affects the overall viscosity and flow properties of the uncured flowable resin composite.^28,30^ A less optimal filler dispersion could hinder the ability of the flowable composite to form thin films under pressure.

Other chemical considerations may influence different materials with dual cure mechanisms. For example, the difference in the curing kinetics is influenced by the reactivity of the monomer’s functional groups between TEGDMA and IBOMA. TEGDMA’s faster rate of polymerization due to its dual methacrylate groups leads to an earlier gelation point during the curing process, which reduces the material’s ability to flow and conform to minimal thicknesses before becoming rigid. Conversely, IBOMA’s slower polymerization rate allows for extended flow under pressure, which contributes to thinner films.

Water sorption and solubility have been reported to affect the structure and function of flowable resin composites. W_sl_ in resin-based materials is a diffusion-controlled process that occurs mainly in the resin matrix.^31^ TEGDMA is known to be more hydrophilic than IBOMA; due to the fact that TEGDMA is a linear monomer, while IBOMA is a bicyclic compound side chain. In addition, the crosslink density of a resin-based material can also interfere with its W_sp_ and W_sl_. Decreases in the hydrophilicity and increases in the crosslink density of the flowable composites might reduce their W_sp_ and W_sl_.^32,33^ These findings help explain the significantly lower W_sp_ and W_sl_ mean values observed in the present study for IBOMA and TEG-IBO, when compared with TEGDMA (Table 2). When compared with TEGDMA, IBOMA is highly hydrophobic, has lower degree of conversion, and forms fewer crosslinks among the polymer chains.^31-34^These findings might account for significantly higher HR mean values observed for IBOMA and TEG-IBO.

As observed, IBOMA reduced the DC of the flowable resin composites (Table 1), which led to a significant reduction in FM, FS, and KH and a significant increase in HR mean values (Tables 2 and 3). These results seem to be explained by IBOMA’s lack of crosslinking ability.^31^ However, this hypothesis is based on the known limitation of IBOMA’s of only having a single methacrylate functional group (Figure 1). In contrast, when compared with IBOMA, TEGDMA is known as a conventional crosslinking monomer.^15^ The results showed that the crosslinking did not seem to be affected when combining IBOMA and TEGDMA, thus contributing to lower PSS without negatively affecting the main mechanical properties.

TEG-IBO showed a DC mean value significantly higher than that of IBOMA and lower than that of TEGDMA (Table 1). This increase in the DC of TEG-IBO seemed to be enough to improve its KH and FS, like those obtained for TEGDMA. In addition, TEG-IBO showed PSS, Rp_max_, and FT mean values significantly lower than that observed for TEGDMA. These properties are crucial for formulating resin-based materials.^7^ These results are in accord with previous studies, which report IBOMA as a promising monomer for the development of novel resin-based materials.^7,15-18^

It is important to state that most of these studies^7,16,18^did not add filler particles in the resin formulations tested, and the addition of filler particles does play a role in the DC as well as other physical properties of resin materials. Conversely, our study evaluated the possibility of reducing TEGDMA concentration using IBOMA in formulations containing filler particles. Thus, the addition of this component is essential to develop new resin-based composite formulations, especially when the main objective is to directly or indirectly reduce polymerization shrinkage and water sorption /solubility. Thus, further studies should consider testing additional properties using formulations containing filler particles.

Despite these mixed results, the use of IBOMA as a diluent monomer appears promising given its ability to reduce undesirable effects like polymerization shrinkage stress and water sorption. Moreover, it remains uncertain whether the statistical differences identified would translate into clinically significant improvements in the performance of these flowable composites. The complexity of the oral environment and the dynamic stresses placed on dental materials in a clinical setting introduce variables that *in vitro* tests cannot fully replicate. Therefore, while this study provides valuable insights into the potential advantages of incorporating IBOMA over TEGDMA in resin-based materials, the actual impact on clinical outcomes, such as the longevity and reliability of restorations, requires further investigation. Future studies, particularly those involving clinical trials (or even laboratory studies involving aging), are essential to determine whether the benefits observed in the laboratory setting will replicate in the improved performance of resin-based materials in dental practice.

## Conclusion

Within the limitations of this study, it was possible to conclude that IBOMA combined with TEGDMA effectively reduced the polymerization shrinkage stress and decreased the water sorption and solubility of flowable resin composites without significantly impacting most of their physicochemical properties. Overall, the tested diluent monomer shows potential for use in flowable resin composites, although further refinement and investigation are still necessary.
